# Identification of unusual phospholipids from bovine heart mitochondria by HPLC-MS/MS

**DOI:** 10.1194/jlr.RA120001044

**Published:** 2020-09-30

**Authors:** Junhwan Kim, Charles L. Hoppel

**Affiliations:** 1 Center for Mitochondrial Diseases, Case Western Reserve University, Cleveland, OH, USA; 2 Department of Pharmacology, Case Western Reserve University, Cleveland, OH, USA; 3 Department of Medicine, Case Western Reserve University, Cleveland, OH, USA

**Keywords:** collision-induced dissociation, acyl chain, ether phospholipids, cardiolipin, odd-numbered acyl chain, high-performance liquid chromatography-tandem mass spectrometry, lipidomics

## Abstract

Phospholipids, including ether phospholipids, are composed of numerous isomeric and isobaric species that have the same backbone and acyl chains. This structural resemblance results in similar fragmentation patterns by collision-induced dissociation of phospholipids regardless of class, yielding complicated MS/MS spectra when isobaric species are analyzed together. Furthermore, the presence of isobaric species can lead to misassignment of species when made solely based on their molecular weights. In this study, we used normal-phase HPLC for ESI-MS/MS analysis of phospholipids from bovine heart mitochondria. Class separation by HPLC eliminates chances for misidentification of isobaric species from different classes of phospholipids. Chromatography yields simple MS/MS spectra without interference from isobaric species, allowing clear identification of peaks corresponding to fragmented ions containing monoacylglycerol backbone derived from losing one acyl chain. Using these fragmented ions, we characterized individual and isomeric species in each class of mitochondrial phospholipids, including unusual species, such as PS, containing an ether linkage and species containing odd-numbered acyl chains in cardiolipin, PS, PI, and PG. We also characterized monolysocardiolipin and dilysocardiolipin, the least abundant but nevertheless important mitochondrial phospholipids. The results clearly show the power of HPLC-MS/MS for identification and characterization of phospholipids, including minor species.

Phospholipids are important components of mitochondrial membranes and maintain intactness of membranes and proper mitochondrial function. Generally, phospholipids are composed of a DAG moiety attached to a phosphate group, which in turn is connected to various head groups. These head groups determine the class of phospholipids, whereas the acyl chains determine the species of phospholipids within a class. An exception is cardiolipin (CL), which contains two DAG phosphate moieties linked by another glycerol molecule. Different combinations of the head groups and the acyl chains give rise to numerous isobaric and isomeric species of phospholipids.

MS has been widely used to characterize the structure of phospholipids. Previous studies have identified common acyl chains of phospholipids. For example, stearic acid (18:0), palmitic acid (16:0), and myristic acid (14:0) are major acyl chains found at the sn-1 position of phospholipids, whereas linoleic acid (18:2), oleic acid (18:1), arachidonic acid (20:4), and docosahexaenoic acid (22:6) are found at the sn-2 position. Other fatty acids, such as (16:1), (18:1), (22:3), (20:4), and (20:5), are also often found in phospholipids; typically, the more saturated fatty acids are linked at the sn-1 position and unsaturated fatty acids at the sn-2 position (1).

Using this concept and commonly observed acyl chains, assignments of peaks based on their masses are generally accepted for global lipidomics (2–4). However, this type of analysis does not differentiate between isomeric species and thus neglects the importance of individual species. Furthermore, there is a chance for misidentification between isobaric species, especially when unusual or unexpected species such as ether phospholipids or phospholipids with odd-numbered acyl chains are present. Ether phospholipids, including plasmanyl and plasmenyl phospholipids, contain an ether akyl chain at the sn-1 position. Although the presence of ether phospholipids in mitochondria was reported previously (5), the presence of ether phospholipids in mitochondria is not generally recognized (6). Furthermore, ether-linked PS species have not been observed in mammalian mitochondria.

Recently, we reported an HPLC-MS method for class separation of phospholipids (7). In the current study, we applied the same HPLC conditions for qualitative analysis of phospholipids from bovine heart mitochondria (BHMs) by collision-induced dissociation (CID). Our data markedly differ in the assignments of phospholipid classes and species compared with those recently made in MALDI-TOF lipidomics of BHMs (2). In brief, they labeled six species of PS, which were not contained in our PS class, and we identified these as ether-linked PE. Moreover, the major PS species we detected were not reported by them. The reason for these discrepancies was that we removed interference from isobaric species by chromatographic separation before mass analysis, thus providing clean MS/MS spectra.

Two major fragment ions generated under the conditions used were carboxylate anions of free fatty acids and ions from the neutral loss of free fatty acids. The latter contain the monoacylglycerol (MAG) backbone, which are noted as MAG-like ions. Because of the preferential cleavage of acyl chains at the sn-2 position (8, 9), the relative peak intensities of the MAG-like ions have been used to determine the position of acyl chains (10, 11). Herein, we expanded our investigation to biological phospholipids containing various acyl chains and found that, regardless of their classes and acyl chains, the peaks corresponding to MAG-like ions containing more saturated acyl chains were always more intense than those corresponding to MAG-like ions containing less saturated acyl chains, consistent with the typical structure of phospholipids (1). These results further confirm that the relative intensity of MAG-like ions can be used to determine the position of acyl chains. We also demonstrate the use of MAG-like ions to differentiate the typical DAG phospholipids from ether phospholipids using the distinct fragment patterns by CID, as previously identified (12, 13).

Chromatographic separation also enabled us to characterize unusual species such as ether-linked PS and phospholipids containing odd-numbered acyl chains, particularity in CL. More importantly, we characterized seven species of monolysocardiolipin (MLCL) and two structural isomers of dilysocardiolipin (DLCL), which previously have not been identified. The results clearly show that HPLC-MS/MS is a powerful tool for analysis of phospholipids.

## MATERIALS AND METHODS

### Chemicals and materials

BHMs were purchased from Abcam (Cambridge, MA). HPLC-grade solvents and formic acid were obtained from Fisher Scientific (Pittsburgh, PA). Standard phospholipids of biological origins and synthetic phospholipids, PE(16:0/18:1), PS(16:0/18:2), and PC(18:0/22:6), were obtained from Avanti Polar Lipids (Alabaster, AL). Butylated hydroxytoluene, ammonium formate, and 1,2-dipalmitoyl-*sn*-glycero-3-phospho-*N*-methylethanolamine (PME; used as the internal standard) were purchased from 3B Scientific Corp. (Libertyville, IL). Prepacked silica gel SPE columns were purchased from Biotage (Charlotte, NC). Reagent grade water was prepared using a Milli-Q reagent water system (Millipore, Inc., Bedford, MA).

### Extraction of mitochondrial phospholipids

Extraction and isolation of phospholipids were performed as previously reported (7). Briefly, 60 μg of mitochondria in 50 μl of potassium phosphate buffer (100 mM, pH 7.4) were extracted by 950 μl of a solution of CHCl_3_:methanol, 2:1 containing butylated hydroxytoluene (2 mM). The extracted lipid mixture was separated to harvest the phospholipid fraction by solid-phase extraction using prepacked silica gel SPE columns. The phospholipid fraction was reconstituted in 200 μl of eluent A containing IPA:TBME:ammonium formate (34:17:5), and 25 μl were injected into the HPLC.

### Normal-phase HPLC-MS and MS/MS

The HPLC conditions to separate each class of phospholipid were reported previously (7). A nucleosil diol column (5 μm, 3 × 250 mm) from Macherey-Nagel (Duren, Germany) was used. Eluent A contained IPA:TBME:ammonium formate (340:170:50) and eluent B contained methanol. Aqueous ammonium formate (pH ∼2.5) was prepared by dissolving 295 mg of ammonium formate and 2 ml of formic acid in 50 ml of water. The gradients used for the 45 min chromatogram were as follows: 100% A for 20 min, 100% A to 20% A over 10 min, 20% A for 6 min, 20% A to 100% A over 1 min, and hold 100% A for 6 min. The flow rate was 0.3 ml/min, and the column temperature was 30°C. MS and MS/MS data were obtained with a Thermo LTQ Velos spectrometer (Thermo Scientific, San Jose, CA) operated in the negative ion mode. The capillary temperature was 300°C. The MS scan was performed with a spectral range of 1,400–1,600 Da, and an MS/MS scan was performed on the most intense ion in the full-scan spectrum. The concentrations of each individual class of phospholipids were determined using standard curves and an internal standard (7). The question of whether the initial acidic condition of eluent A and use of a silica column could hydrolyze ether phospholipids was examined. The standard PE used contains a significant amount of ether phospholipids; we did not detect LPE in the standard curves. Additionally, LPC was not detected in the standard curves for PC.

## RESULTS

### Quantitation of phospholipids

**Fig. 1.**
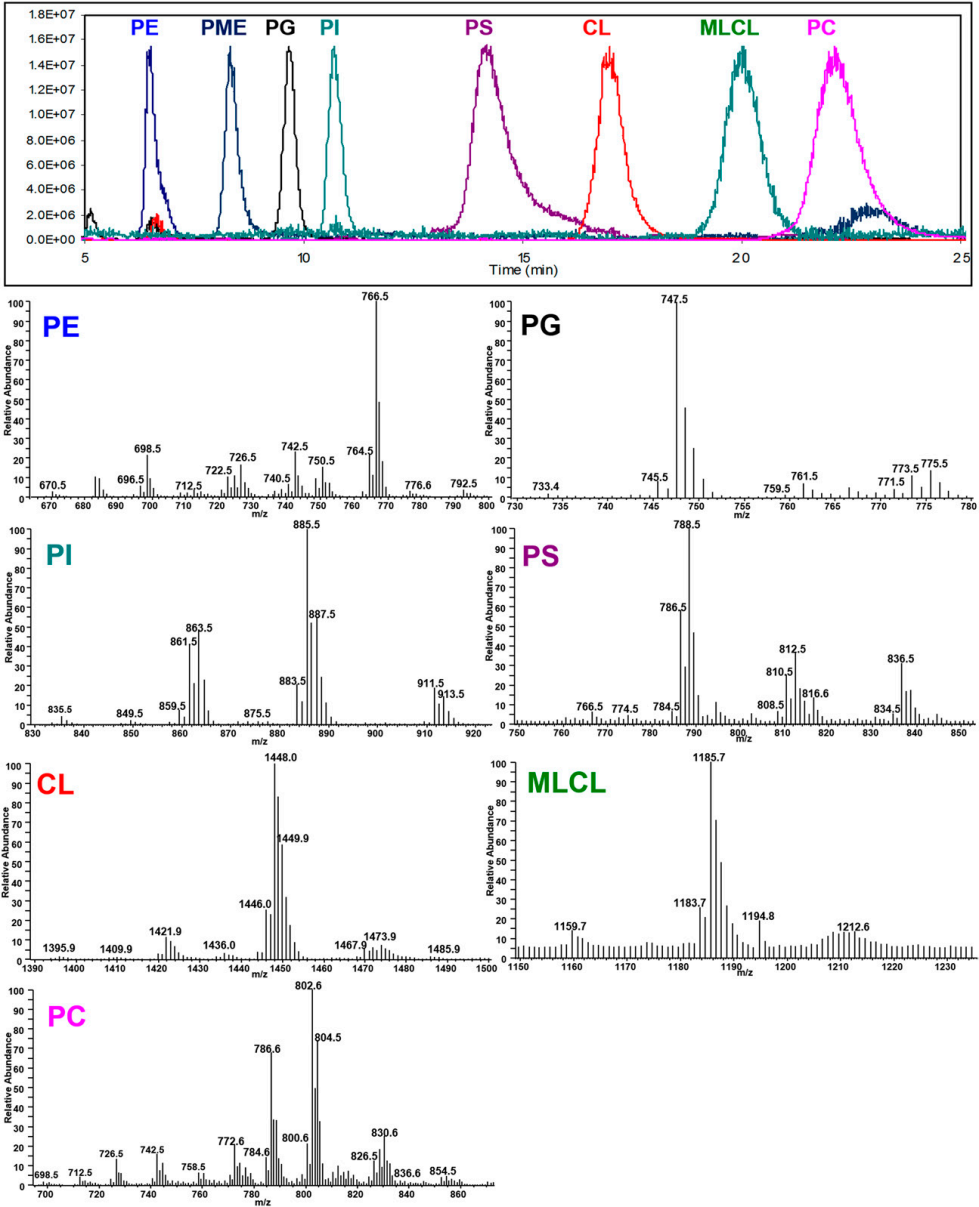
Normalized ion chromatograms of PE, PG, PI, PS, CL, MLCL, and PE from BHMs and PME, the internal standard (upper panel) and mass spectra of individual classes of phospholipids (lower panels). The ion chromatograms were generated using the most abundant species in each class of phospholipids: PE (blue), 766–768; PG (black) 747–749; PI (teal) 885–887; PS (violet), 788–780; CL (red), 1,447–1,450; MLCL (green), 1,185–1,187; and PC (pink), 802–804. The ion chromatogram of PS was limited to its retention time because of high background in the PC and PE regions.

The ion chromatogram and mass spectra of seven classes of phospholipids (PE, PG, PI, PS, CL, MLCL, and PC) from BHMs and PME, the internal standard, are shown in **Fig. 1**. The normalized ion chromatograms in Fig. 1 were generated using the mass of the most intense species in each class of phospholipid.

Of the seven classes of phospholipids, six were quantified using standard curves and the internal standard as previously reported (7). The mass ranges used for quantitation were *m/z* 670–796 (PE), *m/z* 733–777 (PG), *m/z* 835–917 (PI), *m/z* 1,393–1,500 (CL), *m/z* 1,159–1,220 (MLCL), and *m/z* 784–864 (PC). The concentrations of each class of phospholipid from BHMs are shown in supplemental Table S1 and are compared with the concentrations of phospholipids from mitochondria of mouse heart, rat heart, and rat skeletal muscle determined using the same method.

### MS/MS fragmentation patterns of synthetic phospholipids

**Fig. 2.**
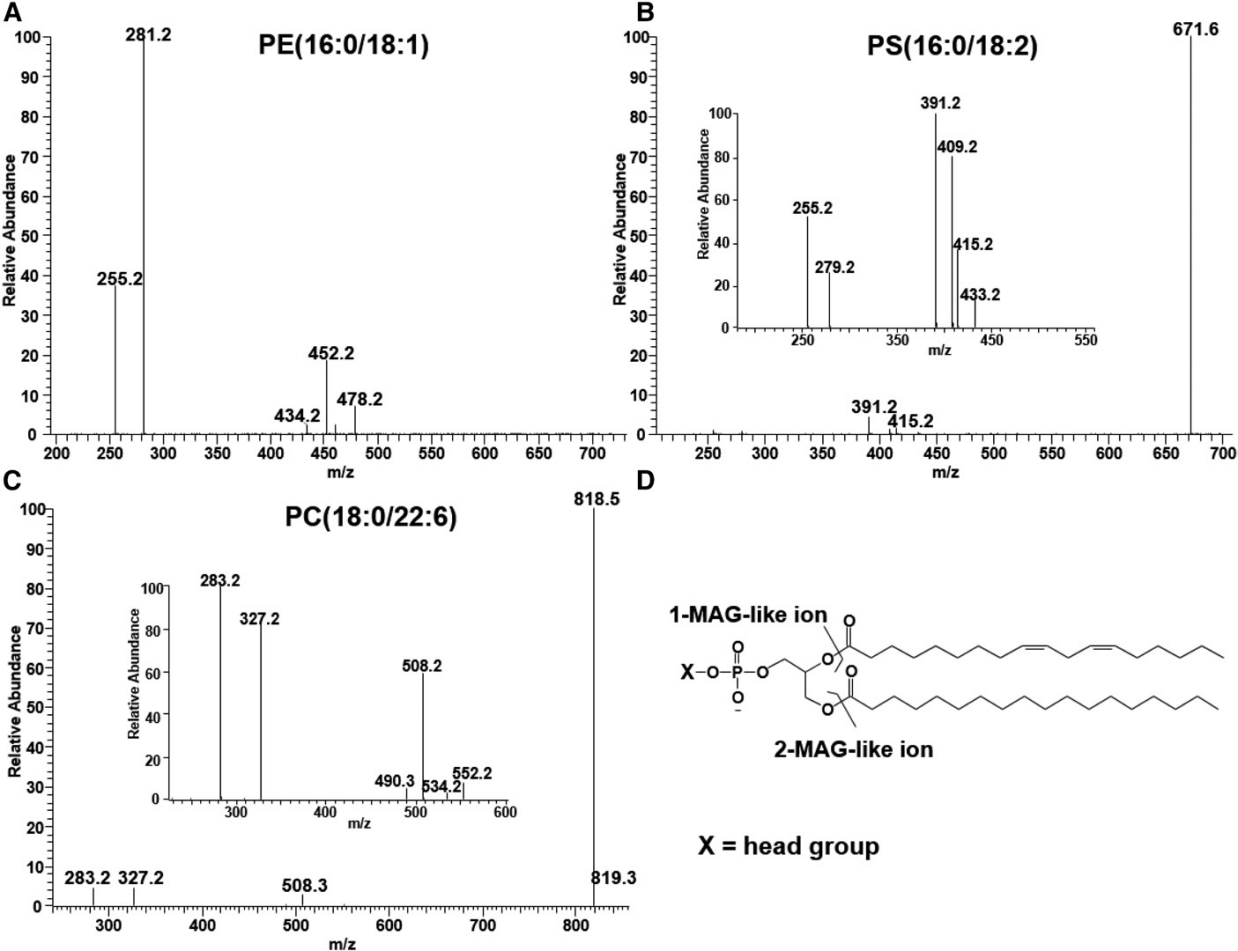
The MS/MS spectra of standard synthetic phospholipids PE(16:0/18:1), *m/z* 716.5 (A), PS(16:0/18:2), *m/z* 758.5 (B), and PC(18:0/22:6), *m/z* 878.5 (C), obtained in the negative ion mode, and fragmentation of phospholipids leading to1-MAG-like ions and 2-MAG-like ions (D). The MS/MS peaks corresponding to 1-MAG-like ions, *m/z* 452/434 (A), *m/z* 409/391 (B), and *m/z* 508/490 (C), are more intense than the peaks corresponding to 2-MAG-like ions, *m/z* 478/460 (A), *m/z* 433/414 (B), and *m/z* 552/534 (C), for all three species.

To examine the fragmentation patterns of phospholipids, three synthetic standard phospholipids, PE(16:0/18:1), PS(16:0/18:2), and PC(18:0/22:6) in eluent A were analyzed by direct infusion (**Fig. 2**). The MS/MS spectrum of PE(16:0/18:1), *m/z* 716.5, shows peaks that correspond to MAG-like ions and carboxylate anions (Fig. 2A). The peak at *m/z* 452 is derived from the loss of 18:1 as a ketene and the peak at *m/z* 434 from the loss of the same acyl chain as carboxylate anion, as discussed in previous reports (11, 14). Likewise, the peaks at *m/z* 478 and 460 are derived from the loss of 16:0 as ketene and carboxylate anion, respectively. The peaks at *m/z* 452 and 434 correspond to a MAG-like ion containing 16:0 and are noted as 452/434, and peaks at *m/z* 478 and 460 correspond to a MAG-like ion containing 18:1 as 478/460. The peaks at 255 and 281 correspond to carboxylate anions, 16:0 and 18:1, respectively. Assignments of the peaks are consistent with previous reports (14, 15).

Figure 2B shows the MS/MS of PS(16:0/18:2) after collision activation of *m/z* 758.5. The most intense peak at *m/z* 671 is derived from the loss of the serine head group. Although this peak does not give any information on the acyl chain composition, the mass loss of 87 Da is a signature fragmentation of PS species (11, 13). The rest of the peaks are assigned in the same way used for those of the PE species. The peaks at *m/z* 409/391 correspond to a MAG-like ion with 16:0, and the peaks at *m/z* 433/415 to a MAG-like ion with 18:2. The peaks at *m/z* 255 and 279 correspond to carboxylate anions, 16:0 and 18:2, respectively.

Finally, we analyzed standard PC(18:0/22:6), which is detected as a formate adduct with *m/z* 878.5 in the negative ion mode. The most intense peak at *m/z* 818 is derived from demethylation of the choline head group (Fig. 2C). This mass loss of 60 Da is a characteristic fragment of PC formate adducts as previously shown (16). Assignments of the remaining peaks are made as they were for the PS and PE species; peaks at *m/z* 283 and 327 correspond to 18:0 and 22:6, the peaks at *m/z* 508/490 correspond to a MAG-like ion containing 18:0, and the peaks at *m/z* 552/534 correspond to a MAG-like ion containing 22:6. As expected, all three classes of phospholipids provided a similar pattern of fragmentation by CID.

Previous studies have reported that cleavage at the sn-2 position is preferable to cleavage at the sn-1 position (8, 9). Because of this, the use of relative intensities of carboxylate anions was suggested to determine the positions of acyl chains (17). The problem with this approach is discussed in a review paper (18). Our data also do not support the notion that acyl chain intensities indicate the position of acyl chains (Fig. 2). MS/MS of the PS species generate a more intense peak of the acyl chain from the sn-2 position, while the PC species generates similar intensities for both acyl chains.

In contrast, the peaks corresponding to the MAG-like ions containing acyl chains at the sn-1 position (1-MAG-like ions) are more intense than those containing acyl chains at the sn-2 position (2-MAG-like ions) for all three species. These results are consistent with a previous finding observed from standard phospholipids (10, 11). Except for CL, MLCL, and DLCL, the regiospecificity of acyl chains was assigned following this principle as follows, “PE(acyl chain at sn-1/acyl chain at sn-2),” based on the relative intensities of 1-MAG-like ions and 2-MAG-like ions. However, it is notable that the opposite regioisomers may also be present. Furthermore, because no information about the position of the double bond on alkyl chains can be obtained, we present fatty acids only with total number of carbons and double bonds, e.g., 18:1. We also do not distinguish between plasmanyl and plasmenyl phospholipids. All ether phospholipids are presented as PE(O-16:0/20:4).

### Optimization of CID energy for MS/MS

As shown above, the most intense MS/MS peaks of PS and PC are derived from modification at the head groups. The peaks derived from fragmentation on the head groups, though characteristic for each class, do not provide information about their acyl chains. The low intensities of the MS/MS peaks corresponding to MAG-like ions and carboxylate anions may prevent characterization of minor species in PS and PC in biological samples. Therefore, we varied collision energy for a specific species in each class of standard phospholipid to find the optimal collision energy to produce maximal intensities of MAG-like ions and carboxylate anions by CID (supplemental Fig. S1). The optimal CID energy for maximal MAG-like ions and carboxylate anions is ∼70 eV for PS and PC and ∼35 eV for all of the other phospholipids. Therefore, we applied 70 eV for PS and PC and 35 eV for the rest of phospholipids.

### Analysis of CL

The detailed structure of CL has been extensively studied by MS using free acyl peaks (19, 20). Because of the mass cut-off of the ion trap, MS/MS of CL does not show the mass area for free acyl chains. However, the peaks corresponding to ions containing the DAG backbone (DAG-like ions) and MAG-like ions can be used to identify the four acyl chains. The MS/MS spectra of the well-characterized CL species with *m/z* 1,447.9 and 1,449.9 are shown in supplemental Fig. S2. The detailed assignment on each of the MS/MS peaks was consistent with that discussed previously (21). For our current study, only peaks corresponding to MAG-like ions and DAG-like ions are considered for characterization.

**Fig. 3.**
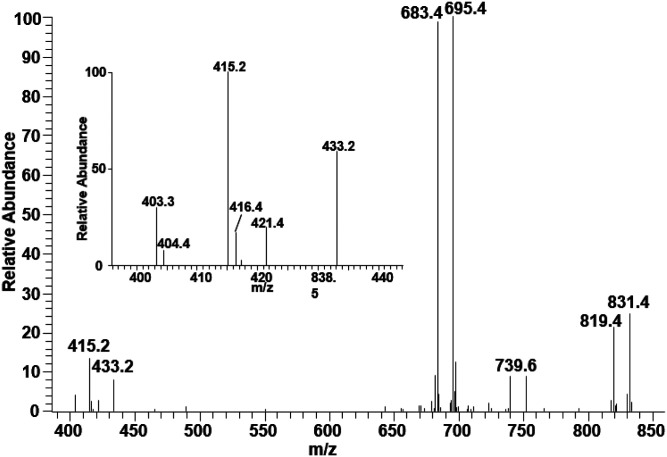
The MS/MS spectrum of a CL molecular species resulting after collisional activation of the [M-H]^−^ ion at *m/z* 1,435.9 obtained in the negative ion mode. The MS/MS peaks at *m/z* 683 and 421/403 are consistent with the structure of CL(18:2_18:2_18:2_17:1).

Using this approach, we also characterized CL containing an odd-numbered acyl chain. The MS/MS spectrum of the MS peak at *m/z* 1,435.9 shows all the characteristic peaks of CL (**Fig. 3**). The peaks corresponding to DAG-like ions, *m/z* 695 and 683, suggest that one DAG-like ion contains two 18:2 and the other DAG-like ion contains 18:2 and 17:1. The two sets of MAG-like ion peaks at *m/z* 421/403 and 433/415 are consistent with the structure of CL(18:2_18:2_18:2_17:1). Conversely, this species may be an ether-linked CL, but because the precursor PG contains 17:1, we infer this to be the CL structure.

Although the peaks corresponding to MAG-like ions are clearly shown in the MS/MS spectra of these three species of CL, they are not always apparent, especially when multiple isomeric species are present. As such, our method cannot clearly distinguish CL species containing odd-numbered chains from their isomeric ether-linked CL species, which may well exist in our samples. However, we confirmed several CL species containing odd-numbered fatty acids using the presence of MAG-like ions generated from the loss of odd-numbered acyl chains. These MAG-like ions would not be generated from ether-linked CL, as our MS/MS method did not break the ether linkage in other ether phospholipid classes. Because we can only confirm the presence of CL species containing odd-numbered acyl chains, our assignment only considers these species. We present the CL structure based on DAG-like ions. For example, CL(18:2_18:2_18:2_18:2) is presented as CL(36:4_36:4) in **Table 1**.

**TABLE 1.** Molecular species of phospholipids from BHMs

Class	*m/z*	Characterized Structure
CL	1,393.9	CL(32:3_36:4), CL(34:3_34:4), CL(32:2_36:5)
	1,395.9	CL(32:2_36:4), CL(34:3_34:3)
	1,397.9	CL(32:2_36:3), CL(34:3_34:2), CL(32:1_36:4), CL(32:3_36:2)
	1,399.9	CL(32:1_36:3), CL(34:1_34:3), CL(34:2_34:2), CL(32:2_36:2)
	1,403.9	CL(34:1_34:1)
	1,405.9	CL(34:1_34:0), CL(33:4_36:4), CL(33:3_36:5)
	1,407.9	CL(33:3_36:4)
	1,409.9	CL(33:2_36:4), CL(33:3_36:3), CL(34:3_35:3)
	1,417.9	CL(34:4_36:5), CL(34:3_36:6)
	1,419.9	CL(34:3_36:5), CL(34:4_36:4)
	1,421.9	CL(34:3_36:4)
	1,423.9	CL(34:3_36:3), CL(34:2_36:4)
	1,425.9	CL(34:2_36:3), CL(34:1_36:4)
	1,427.9	CL(34:1_36:3), CL(34:2_36:2)
	1,433.9	CL(35:4_36:4), CL(35:3_36:5)
	1,435.9	CL(35:3_36:4)
	1,443.9	CL(36:5_36:5), CL(36:4_36:6),
	1,445.9	CL(36:4_36:5)
	1,447.9	CL(36:4_36:4)
	1,449.9	CL(36:4_36:3)
	1,451.9	CL(36:3_36:3), CL(36:4_36:2)
	1,463.9	CL(36:4_37:4)
	1,471.9	CL(36:4_38:6)
	1,473.9	CL(36:4_38:5), CL(36:3_38:6),
	1,475.9	CL(36:4_38:4), CL(36:3_38:5)
	1,479.9	CL(36:4_38:2)
	1,495.9	CL(36:4_40:8)
	1,497.9	CL(36:4_40:7)
MLCL	1,159.6	MLCL(18:2_18:2_16:1)
	1,183.6	MLCL(18:2_18:2_18:3)
	1,185.6	MLCL(18:2_18:2_18:2)
	1,187.6	MLCL(18:2_18:2_18:1)
	1,189.6	MLCL(18:2_18:2_18:0), MLCL(18:1_18:1_18:2)
	1,209.6	MLCL(18:2_18:2_20:4)
	1,201.6	MLCL(18:2_18:2_20:3), MLCL(18:2_18:1_20:4)
DLCL	923.5	DLCL(18:2_18:2)
PE	670.5	PE(O-14:1/18:2)
	698.5	PE(O-16:1/18:2)
	712.5	PE(16:1/18:2)
	714.5	PE(16:0/18:2), PE(16:1/18:1)
	720.5	PE(O-16:1/20:5)
	722.5	PE(O-16:1/20:4)
	724.5	PE(O-18:2/18:2), PE(O-18:1/18:3), PE(O-16:1/20:3)
	726.5	PE(O-18:1/18:2)
	738.5	PE(18:2/18:2), PE(16:0/20:4), PE(16:1/20:5), PE(18:1/18:3)
	740.5	PE(18:1/18:2), PE(18:0/18:3)
	742.5	PE(18:0/18:2)
	748.5	PE(O-18:1/20:5), PE(O-16:1/22:5), PE(O-18:2/20:4)
	750.5	PE(O-18:1/20:4), PE(O-18:2/20:3)
	752.5	PE(O-18:1/20:3), PE(O-18:0/20:4)
	762.5	PE(18:1/20:5), PE(18:2/20:4)
	764.5	PE(18:0/20:5), PE(18:1/20:4)
	766.5	PE(18:0/20:4)
	776.5	PE(O-18:1/22:5)
	778.5	PE(O-20:1/20:4), PE(O-18:1/22:4), PE(O-18:0/22:5), PE(O-20:1/20:5)
	780.5	PE(O-20:0/20:4)
	792.5	PE(18:0/22:5)
	794.5	PE(18:0/22:4), PE(20:0/20:4)
PG	733.5	PG(15:0/18:1), PG(16:0/17:1)
	745.5	PG(16:0/18:2)
	747.5	PG(16:0/18:1)
	759.5	PG(17:0/18:2), PG(17:1/18:1)
	761.5	PG(17:0/18:1), PG(16:0/19:1)
	771.5	PG(18:1/18:2), PG(16:0/20:3)
	773.5	PG(18:1/18:1), PG(18:0/18:2), PG(16:0/20:2)
	775.5	PG(18:0/18:1), PG(20:0/16:1)
PI	831.5	PI(16:0/18:3)
	833.5	PI(16:0/18:2)
	835.5	PI(16:0/18:1), PI(18:0/16:1)
	849.5	PI(17:0/18:1), PI(18:0/17:1)
	857.5	PI(16:0/20:4)
	859.5	PI(16:0/20:3), PI(18:0/18:3), PI(18:1/18:2)
	861.5	PI(18:0/18:2)
	863.5	PI(18:0/18:1)
	871.5	PI(17:0/20:4)
	873.5	PI(17:0/20:3), PI(18:0/19:3)
	875.5	PI(18:0/19:2)
	877.5	PI(18:0/19:1)
	883.5	PI(18:0/20:5), PI(18:1/20:4)
	885.5	PI(18:0/20:4)
	887.5	PI(18:0/20:3)
	889.5	PI(18:0/20:2)
	891.5	PI(18:0/20:1)
	901.5	PI(18:0/ 19:3)
	911.5	PI(18:0/22:5)
	913.5	PI(18:0/22:4)
	915.5	PI(18:0/22:3)
PS	758.5	PS(16:0/18:2)
	760.5	PS(16:0/18:1), PS(18:0/16:1)
	762.5	PS(18:0/16:0)
	766.5	PS(O-16:1/20:4)
	772.5	PS(17:1/18:1), PS(17:0/18:2)
	774.5	PS(18:0/17:1), PS(17:0/18:1)
	776.5	PS(18:0/17:0)
	780.5	PS(16:0/20:4)
	784.5	PS(18:0/18:3), PS(18:1/18:2)
	786.5	PS(18:0/18:2), PS(18:1/18:1)
	788.5	PS(18:0/18:1)
	794.5	PS(O-18:1/20:4)
	796.5	PS(O-18:1/20:3)
	802.5	PS(18:0/19:1), PS(18:1/19:0)
	808.5	PS(18:0/20:5), PS(18:1/20:4)
	810.5	PS(18:0/ 20:4)
	812.5	PS(18:0/ 20:3)
	816.5	PS(20:0/18:1)
	820.5	PS(O-18:1/22:5)
	836.5	PS(18:0/22:5)
	838.5	PS(18:0/22:4)
	840.5	PS(18:0/22:3), PS(20:0/20:3)
	842.5	PS(22:1/18:1), PS(22:0/18:2)
	844.5	PS(22:0/18:1)
PC	784.5	PC(O-16:1/18:3), PC(O-16:2/18:2)
	786.5	PC(O-16:1/18:2)
	788.5	PC(O-16:1/18:1), PC(O-16:0/18:2)
	800.5	PC(16:0/18:3), PC(16:1/18:2)
	802.5	PC(16:0/ 18:2)
	804.5	PC(16:0/18:1)
	808.5	PC(O-16:1/20:5), PC(O-16:2/20:4)
	810.5	PC(O-16:1/20:4)
	812.5	PC(O-16:1/20:3), PC (O-16:0/20:4), PC(O-18:2/18:2)
	814.5	PC(O-18:1/18:2), PC(O-16:1/20:2), PC(O-16:0/20:3)
	816.5	PC(O-18:0/18:2), PC(O-18:1/18:1)
	818.5	PC(O-18:0/18:1)
	824.5	PC(16:0/20:5)
	826.5	PC(16:0/20:4), PC(18:2/18:2)
	828.5	PC(16:0/20:3), PC(18:1/18:2), PC(18:0/18:3)
	830.5	PC(18:0/18:2)
	832.5	PC(18:0/18:1)
	834.5	PC(16:0/20:0)
	838.5	PC(O-18:1/20:4), PC(O-16:1/22:4)
	840.5	PC(O-18:1/20:3), PC(O-18:0/20:4)
	852.5	PC(18:01/20:4), PC(18:0/20:5), PC(16:1/22:4), PC(16:0/22:5)
	854.5	PC(18:0/20:4), PC(16:0/22:4)
	856.5	PC(18:0/20:3)
	862.5	PC(O-18:1/22:5), PC(18:0/20:0)

### Analysis of MLCL and DLCL

**Fig. 4.**
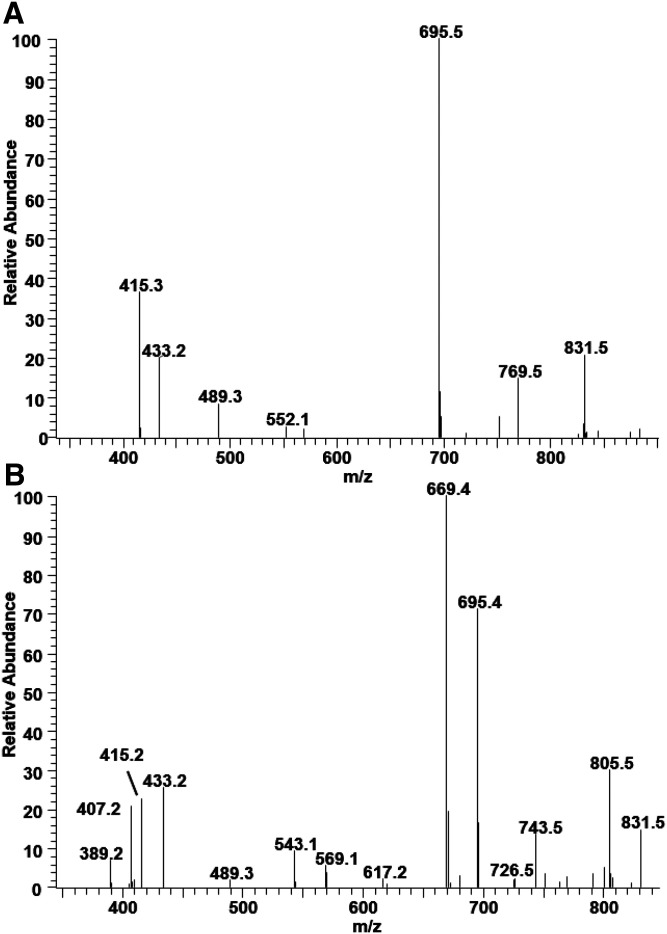
The MS/MS spectrum of MLCL molecular species resulting after collisional activation of the [M-H]^−^ ions at *m/z* 1,185.6 (A) and 1,159.6 (B). The MS/MS peaks at *m/z* 433/415 (A) and *m/z* 433/415 and 407/389 (B) are consistent with the structures of MLCL(18:2_18:2_18:2) and MLCL(18:2_18:2_16:1), respectively.

Because of low abundance, MLCL is not readily detected in global lipidomics. However, we not only detect MLCL but also can characterize several MLCL species by MS/MS. The most abundant MLCL has *m/z* 1,185.6, suggesting that the species contains three 18:2s. The MS/MS spectrum of this species is similar to that of CL(18:2_18:2_18:2_18:2) but only with more intense MAG-like ion peaks at *m/z* 433/415 (**Fig. 4A**). This higher intensity allows identification of all three acyl chains of MLCL species. Using the same peak assignment used for CL(18:2_18:2_18:2_18:2), this MLCL species with *m/z* 1,185.6 is characterized as MLCL(18:2_18:2_18:2), as previously reported (22).

The MS/MS spectrum of the MLCL with *m/z* 1,159.6 is shown in Fig. 4B. The peaks at *m/z* 433/415 and 407/389 show that this species has two types of acyl chains, 18:2 and 16:1. With peaks corresponding to a DAG-like ion, 669 and 695, the species is characterized as MLCL(18:2_18:2_16:1). In a similar manner, acyl chain compositions of other MLCL species have been characterized (Table 1).

**Fig. 5.**
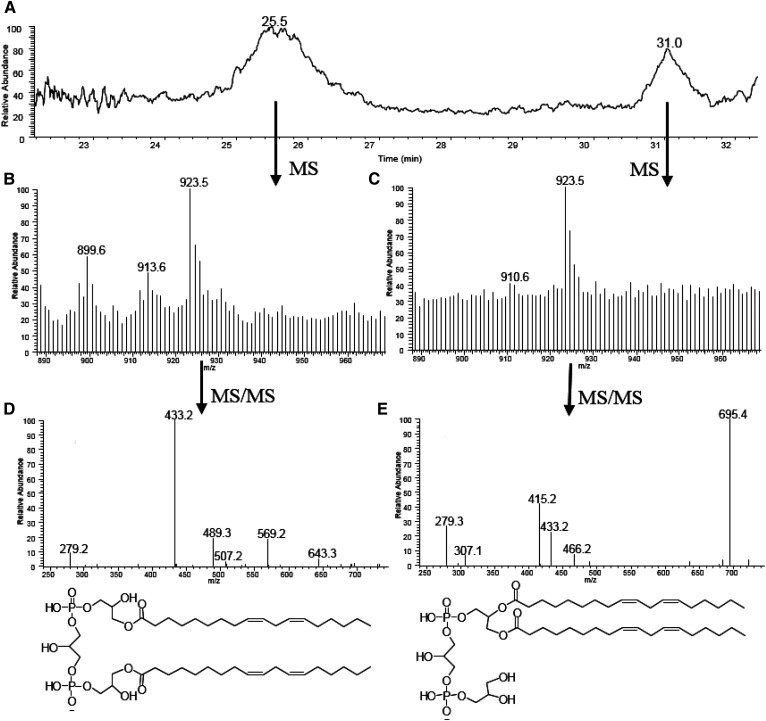
Ion chromatogram of DLCL with *m/z* 923–925 (A), the MS spectra of ion chromatogram peak eluting at 25.5 min (B) and at 31.0 min (C), the MS/MS spectra of the peak at *m/z* 923.5 at 25 min (D) and at 31 min (E). The proposed structures based on MS/MS spectra are shown (the acyl position of DLCL eluting at 25 min and the position of double bonds in both structures are arbitrarily assigned).

DLCL is another metabolite of CL and one of the least abundant phospholipids. The presence of DLCL has been reported in tafazzin knockdown mice in which MLCL and DLCL are increased due to the absence of tafazzin (23). In our study, we show the presence of two types of DLCL by MS/MS from normal BHMs. In the ion chromatogram of *m/z* 923–925, two peaks were noted between 25 and 32 min (**Fig. 5**); DLCL(18:2_18:2), produced from bovine CL by phospholipase A2, is observed at 24–25 min. MS spectra from both chromatographic peaks show the *m/z* 923.5, but are close to the detection limit. However, the MS/MS spectra of both peaks eluting at 25 and 31 min clearly show the characteristic fragmentation pattern of DLCL.

The MS/MS spectrum of the species eluting at 25 min is the same as that previously reported with standard DLCL generated by phospholipase A2 (22), proving that this is a DLCL species with each acyl chain on a different glycerol phosphate moiety. The MS/MS spectrum of the species eluting at 30 min is similar to the MS/MS spectrum of MLCL. The peak at 695 supports the notion that this species has both acyl chains on the same glycerol phosphate moiety. The peak at *m/z* 279 corresponding to 18:2 in both MS/MS spectra also supports the proposed structure of DLCL.

### Analysis of PE

Using the fragmentation pattern observed with the synthetic standard PE, the MS and MS/MS spectrum of PE from BHMs was examined. The MS/MS spectrum of the most intense PE species at *m/z* 766.5 is shown in supplemental Fig. S3A. The peaks at 303 and 281 show that the species contains two acyl chains, 18:0 and 20:4 (the peak at 259 is derived from 303). The peaks at *m/z* 480/462 correspond to a MAG-like ion containing 18:0, and the peak at *m/z* 500 corresponds to a MAG-like ion containing 20:4. The greater intensity of 480 compared with 500 indicates that 18:0 is at the sn-1 position and 20:4 is at the sn-2 position. The fragmentation pattern is consistent with the previous report (14).

The MS/MS spectrum of the MS peak at *m/z* 764.5 is more complicated, suggesting that multiple species contribute to this MS peak (supplemental Fig. S3B). Two MAG-like ion peaks identified at *m/z* 480/462 and 478 and corresponding carboxylate anions at *m/z* 283 and 81 indicate that there are at least two major species containing 18:0 and 18:1 at the sn-1 position. With matching carboxylate anion peaks at *m/z* 301 and 303 and weak MAG-like ion peaks at *m/z* 500 and 498, these species were characterized as PE(18:0/20:5) and PE(18:1/20:4), contributing to the MS peak at *m/z* 764.5.

**Fig. 6.**
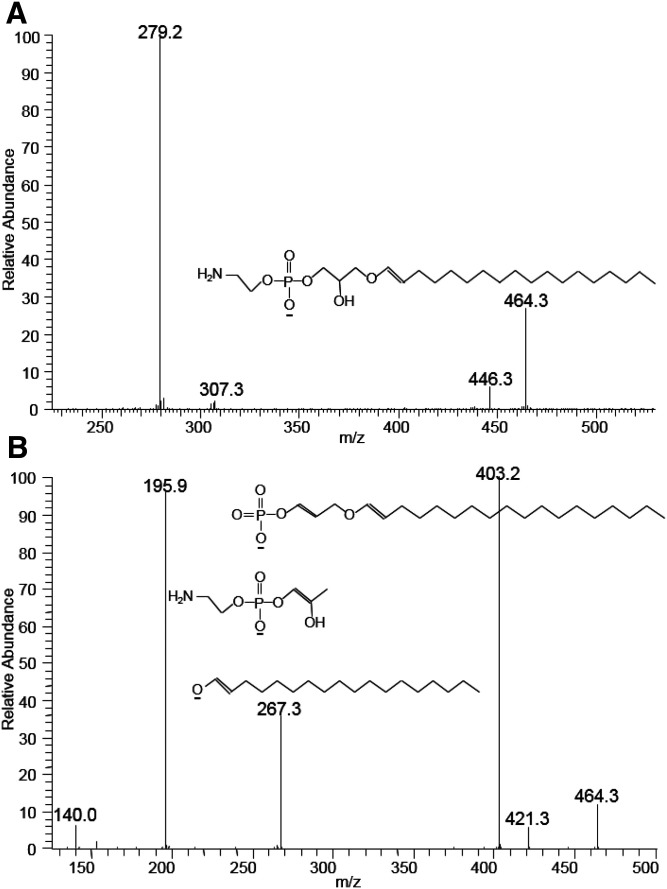
The MS/MS spectrum of a PE molecular species resulting after collisional activation of the [M-H]^−^ ion at *m/z* 726.5 (A) and MS/MS/MS spectrum of the fragmented ion at *m/z* 464 with proposed structures of fragmented ions (B) (the position of double bonds is arbitrarily assigned). The absence of MS/MS peaks corresponding to the 2-MAG-like ion (A) and corresponding MS/MS/MS peaks at *m/z* 403, 267, and 196 of the MS/MS peak at *m/z* 464 (B) are consistent with the structure of PE(O-18:1/18:2).

The observed MS peaks at *m/z* 750.5, 726.5, 724.5, 722.5, and 698.5 indicate that these species have unusual acyl chain composition because combinations of common acyl chains do not account for these ions. The detailed structure was further analyzed. The MS/MS spectrum of the ion at 726.5 is shown in **Fig. 6A**. Unlike other PE species, the MS/MS of this species shows peaks corresponding to only one MAG-like ion (*m/z* 464/446) and one carboxylate anion (*m/z* 279). This MS/MS fragmentation pattern is consistent with ether phospholipids, as previous reported (12, 15, 24). Therefore, the species with *m/z* 726.5 was characterized as an ether-linked PE, PE(O-18:1/18:2). To verify this, the structure of the MAG-like ion with *m/z* 464 was further analyzed by MS/MS/MS (Fig. 6B) (12).

Ether-linked PEs also contain isomers. The MS/MS of the MS peak at *m/z* 724.5 shows a more complicated fragmentation pattern (supplemental Fig. S4); The peaks at *m/z* 436, 462, and 464 indicate at least three major MAG-like ions containing the ether bonds of 16:0, 18:0, or 18:1. With matching acyl chains at the sn-2 positions, as shown by the peaks at *m/z* 305, 279, and 277, three species were characterized as PE(O-16:1/20:3), PE(O-18:2/18:2), and PE(O-18:1/18:3). Likewise, the rest of the PE species were characterized, and the results are summarized in Table 1.

### Analysis of PG

**Fig. 7.**
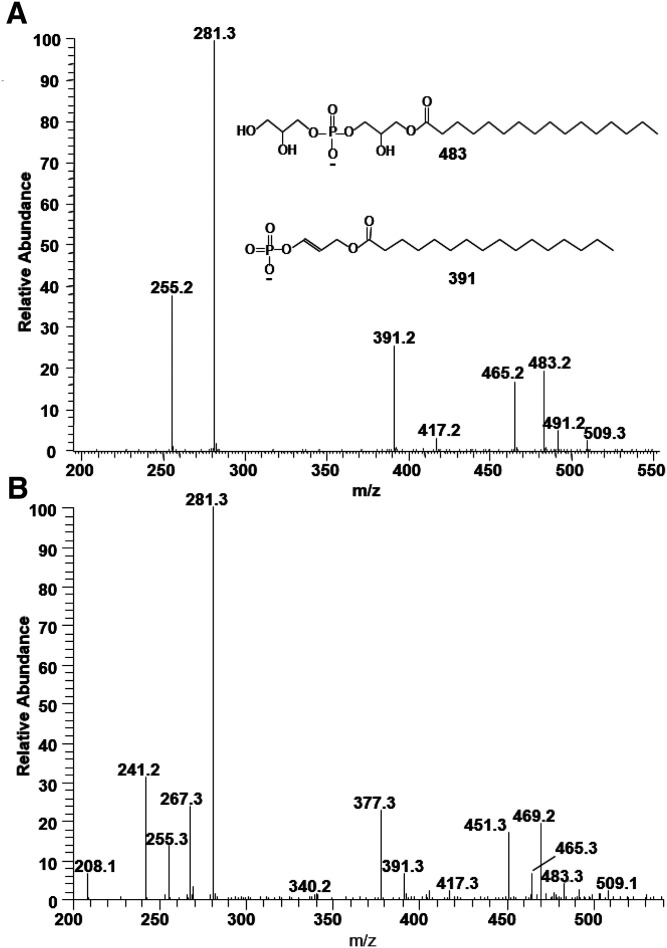
The MS/MS spectrum of PG molecular species resulting after collisional activation of the [M-H]^−^ ions at *m/z* 747.5 (A) and 733.5 (B). The more intense MS/MS peaks corresponding to the 1-MAG-like ion at *m/z* 391 and 483/465 are consistent with the structure of PG(16:0/18:1) (A), and two sets of 1-MAG-like ions, *m/z* 377 and 469/451 and *m/z* 391 and 483/465, are consistent with the structures of PG(15:0/18:1) and PG(16:0/17:1) (B).

The fragmentation pattern of BHM PG is similar to that of PE, generating carboxylate anions and MAG-like ions. However, CID applied to PG generates additional MAG-like ions derived from fragmentation on the head groups. **Figure 7A** shows the MS/MS spectrum of the MS peak at *m/z* 747.5, the most abundant species of PG. The peaks at *m/z* 391 and 483/465 correspond to a MAG-like ion containing 16:0, and the peaks at *m/z* 417 and 509/491 correspond to a MAG-like ion containing 18:1. Matching carboxylate anion peaks are also found at *m/z* 255 and 281. As shown for PE, the more intense MAG-like ion contains 16:0, indicating that the structure is PG(16:0/18:1) with 16:0 at the sn-1 position. The MS/MS spectrum is essentially the same as that of standard PG(16:0/18:1) (11).

Likewise, we characterized several isomeric species. For example, the peak at *m/z* 771.5 is composed of PG(18:1/18:2) and PG(16:0/20:3), and the peak at *m/z* 773.5 is composed of PG(18:1/18:1), PG(18:0/18:2), and PG(16:0/20:2).

PG containing an odd-numbered acyl chain also is detected. The MS/MS spectrum of the MS peak at *m/z* 733.5 is shown in Fig. 7B. Two sets of MAG-like ions with *m/z* 377 and 469/451 and with *m/z* 391 and 483/465 and matching carboxylate anions with *m/z* 241 and 255 indicate two PG species containing 15:0 and 16:0 at the sn-1 position. The peaks of matching acyl chains with *m/z* 267 and 281 and of weaker MAG-like ion peaks are consistent with the structures of PG(15:0/18:1) and PG(16:0/17:1).

### Analysis of PI

**Fig. 8.**
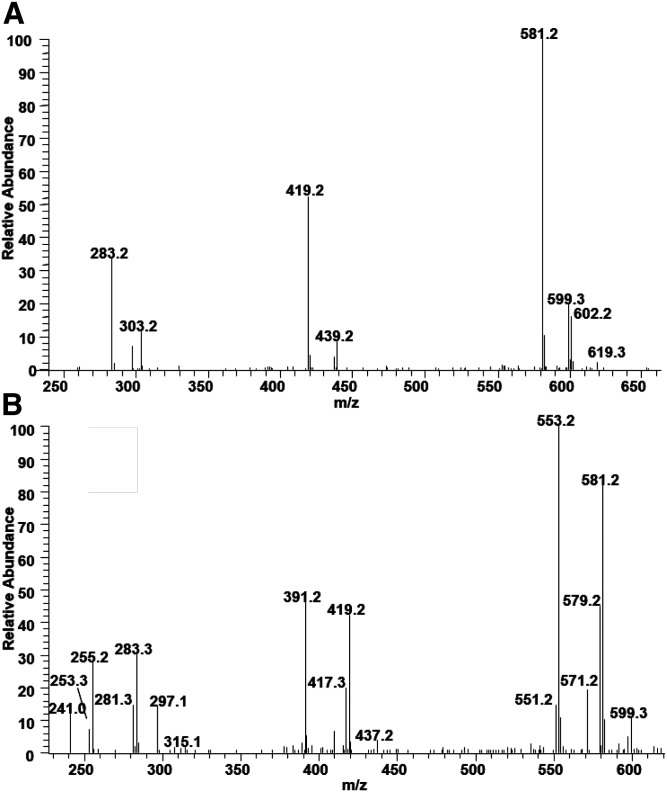
The MS/ MS spectrum of PI molecular species resulting after collisional activation of the [M-H]^−^ ions at *m/z* 885.5 (A) and 835.5 (B). The more intense peaks at *m/z* 599/581 and 419 corresponding to the 1-MAG-like ion are consistent with the structure of PI(18:0/16:1) (A), and the peaks at *m/z* 599/581, 419, and 283 corresponding to one species containing 18:0 and the peaks at *m/z* 571/553, 391, and 255 corresponding to another species containing 16:0 are consistent with the structures of PI(18:0/16:1) and PI(16:0/18:1) (B).

MS/MS spectra of BHM PI also have a similar fragmentation pattern to standard PI reported previously, of carboxylate anions, and multiple MAG-like ions (10). The MS/MS of the most abundant PI (*m/z* 885.5) is shown in **Fig. 8A**. Peaks at *m/z* 599/581 and 419 correspond to a MAG-like ion containing 18:0 and peaks at *m/z* 619/601 and 439 to a MAG-like ion containing 20:4. The relative intensities of these two sets of the peaks support that 18:0 is at the sn-1 position. The peak at 297 is present in all PI species.

The MS/MS spectrum of the peak at *m/z* 835.5 reveals that two major species contribute to this MS peak (Fig. 8B). The peaks at *m/z* 599/581, 419, and 283 correspond to one species containing 18:0 and the peaks at *m/z* 571/553, 391, and 255 correspond to another species containing 16:0. The matching free acyl chain peaks at *m/z* 253 and 281 and MAG-like ion peaks for each species define the species as PI(18:0/16:1) and PI(16:0/18:1).

### Analysis of PS

As shown in the analysis of standard PS, the most intense fragment ion of BHM PS is derived from the cleavage of the head group, resulting in phosphatidic acid moieties with mass loss of 87 Da. This mass loss confirms the identity of PS species (11, 13). The MS spectrum of BHM PS shows six major species of PS with *m/z* 786.5, 788.5, 810.5, 812.5, 836.5, and 838.6 in BHMs. One example of an MS/MS spectrum of PS species with *m/z* 810.5 is shown in supplemental Fig. S5. The peak at *m/z* 723 is derived from the mass loss of 87 Da, characteristic of PS. The more intense peaks at *m/z* 437/419 and 283 indicate that 18:0 is at the sn-1 position; the less intense peaks containing 20:4 at *m/z* 457/439 and 303 prove that this species should be PS(18:0/20:4). Likewise, other species are characterized as PS(18:0/18:2), PS(18:0/18:1), PS(18:0/20:3), PS(18:0/22:5), and PS(18:0/22.4), respectively.

**Fig. 9.**
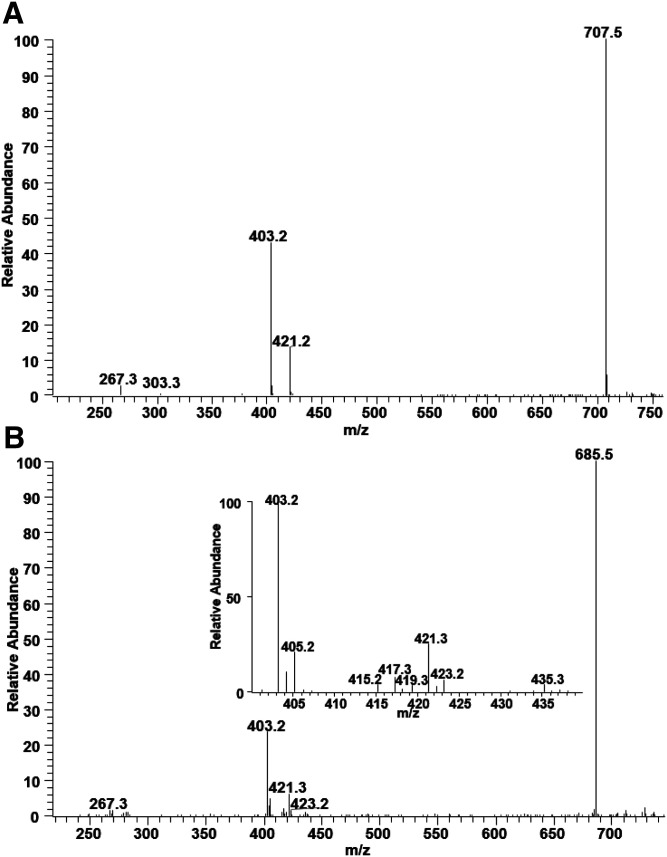
The MS/MS spectrum of PS molecular species resulting after collisional activation of the [M-H]^−^ ions at *m/z* 794.5 (A) and 772.5 (B). The presence of the peaks at *m/z* 421/403 corresponding to the 1-MAG-like ion and the absence of the peaks corresponding to the 2-MAG-like ion are consistent with the structure of PS(O-18:1/20:4) (A). The presence of the peaks at *m/z* 421/403 and 435/417 characterize the major species as PS(17:1/18:1). Minor peaks at *m/z* 423/405 and 415 indicate the presence of PS(17:0/18:2) as a minor isomeric species (B).

We also analyzed minor species of PS. Interestingly, we found several ether-linked PS species and PS species containing odd-numbered acyl chains. MS/MS analysis of the MS peak at 794.5 has the characteristic peak of PS at *m/z* 707, mass loss of 87 Da, confirming that this is a PS species (**Fig. 9A**). The peak at *m/z* 303 shows that one of the acyl chains is 20:4, but peaks corresponding to a MAG-like ion containing 20:4 at *m/z* 457/439 are not apparent. As discussed for PE species, the absence of the peaks corresponding to a 2-MAG-like ion is the characteristic of ether phospholipid. Therefore, this species is identified to be PS(O-18:0/20:4).

Because PS(O-18:0/20:4) (MW: 794.5336) is isobaric with PS(17:1/20:4) (MW: 794.4972), we compared MS/MS of this species with a PS species containing 17:1 to confirm its structure. Figure 9B shows the MS/MS spectrum of a PS species with *m/z* 772.5. The peaks at 421/403 suggest that this species may be PS(O-18:0/18:1) or PS(17:1/18:1). Unlike 794.5, fragmentation of the MS peak at *m/z* 772.5 generates MS/MS peaks corresponding to a 2-MAG-like ion containing 18:1 with *m/z* 435/417, strongly indicating that this species is not an ether phospholipid. The fact that ether phospholipids usually contain highly unsaturated fatty acids also supports the structure of each species. An additional set of peaks at *m/z* 423/405 and 415 show the presence of another isomeric species, PS(17:0/18:2), for the MS peak at *m/z* 772.5 (Fig. 9B).

### Analysis of PC

Under the conditions used, PC is negatively charged by neutralizing the positive charge on the amine group in two different ways: by formation of formate adducts or by demethylation of the quaternary amine to a tertiary amine. These two ionization processes result in a more complicated MS spectrum of PC than the other phospholipids. Because formate adducts are more intense, we analyzed formate adducts of PC species by MS/MS.

CID of formate adducts of BHM PC yields demethylated PCs as the major fragment ions with a mass loss of 60 Da, which is a signature of PC species. The peaks corresponding to MAG-like ions and carboxylate anions further identify the acyl chain composition of individual PC species.

**Fig. 10.**
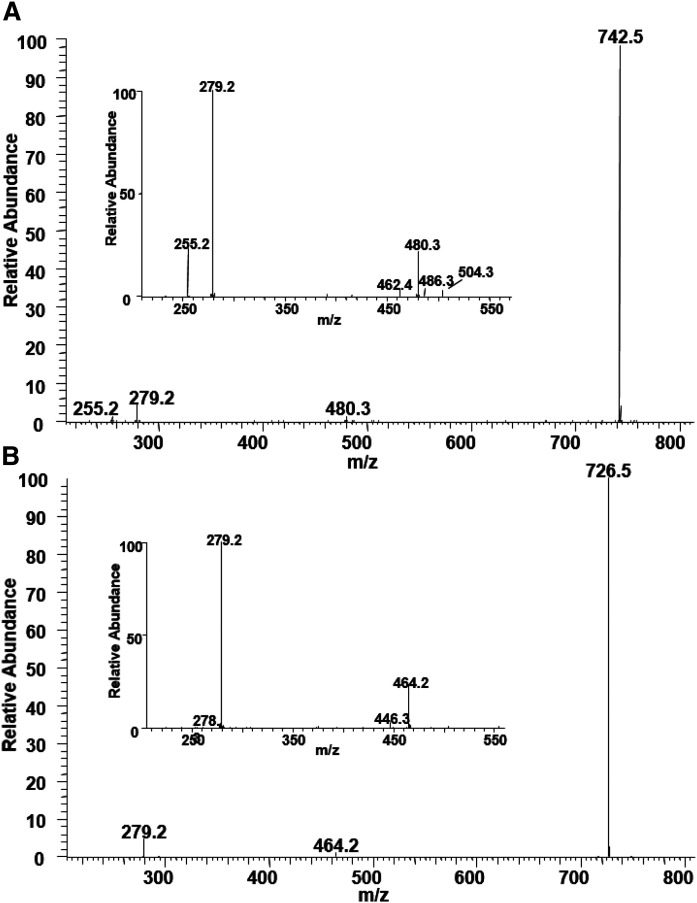
The MS/ MS spectrum of PC molecular species resulting after collisional activation of the [M+HCOO]^−^ ions at *m/z* 802.5 (A) and 786.5 (B). The more intense peaks at *m/z* 480/462 and the less intense peaks at *m/z* 504/486 are consistent with the structure as PC(16:0/18:2) (A), and the peak at *m/z* 464 and the absence of the peaks corresponding to the 2-MAG-like ion are consistent with the structure of PC(O-16:1/18:2).

The MS/MS of the peak at *m/z* 802.5, the most abundant PC species, is shown in **Fig. 10A**. The peak at *m/z* 742 is derived from the mass loss of 60 Da. The more intense peaks at *m/z* 480/462 indicate that 16:0 is at the sn-1 position, and the less intense peaks at *m/z* 504/486 suggest that 18:2 is at the sn-2 position. The presence of the corresponding carboxylate anion peaks at *m/z* 255 and 279 characterizes the structure as PC(16:0/18:2).

BHM PC also contains several ether phospholipid species. The MS/MS spectrum of the peak at *m/z* 786.6 in Fig. 10B shows the peak at *m/z* 464, corresponding to a MAG-like ion containing 16:1 linked by an ether bond. The presence of the peak at *m/z* 279 and the absence of the peaks corresponding to a MAG-like ion with 18:2 at the sn-2 position are consistent with the structure of PC(O-16:1/18:2). Likewise, other PC species, including ether phospholipids, are characterized and listed in Table 1.

## DISCUSSION

MS has been rapidly advanced to the analysis of phospholipids. Because of numerous isobaric and isomeric species with similar fragment patterns by MS/MS, the detailed, yet global, analysis of phospholipids is highly challenging; analysis is aimed for detailed structural information with a limited number or a class of phospholipids, or global analysis provides only incomplete structural information.

Characterization of CL is particularity challenging because of its dimeric structure with four acyl chains that increase the number of isomeric species. The overall number of isomers will be more than what are shown in Table 1, because each DAG-like ions may be composed of several isomers. For some BHM CL species, we clearly identified their acyl chain composition using MAG-like ions, including species containing odd numbered chains (Fig. 3). This is the first clear demonstration of the presence of CL species containing an odd-numbered acyl chain in mammalian mitochondria. It also is noteworthy that CL with *m/z* 1,405.9 is composed of isomers either with all even-numbered species or with an odd-numbered acyl chain (Table 1).

A difficulty with characterizing MLCL and DLCL species is their low abundance. Using MS/MS peaks corresponding to MAG-like ions and DAG-like ions, seven species of MLCLs and two species of DLCLs have been characterized in BHMs. Although DLCL is detected near the detection limit, the class separation reveals two different types of DLCLs with distinct MS/MS spectra, emphasizing the power of HPLC-MS/MS. For further structural information on the acyl chain position of CL, MLCL, and DLCL, synthetic standard species will be needed as well as MS^3^ characterization of DAG-like ions.

The rest of the BHM phospholipids, PE, PC, PG, PI, and PS, compose numerous isomeric and isobaric species, which share similar fragmentation patterns by CID. When analyzed together, these phospholipids result in complicated MS/MS spectra. Additionally, isobaric species may lead to misinterpretation of MS peaks.

A previous study on MALDI-TOF analysis of phospholipids from BHMs assigned peaks with nominal masses of 698.5, 720.5, 722.5, 724.5, 726.5, and 750.5 as PS species (2). In contrast, our data show that these assignments are wrong. We characterized all of these species as ether-linked PE, and we could not detect species with similar masses in PS. Additionally, the major species we characterized in PS were not found in the previous MALDI-TOF study (2). We contend that separation by HPLC eliminates the chance for misassignment between classes because of their different retention times.

Another advantage of HPLC is that chromatography not only separates classes of phospholipids but also spreads individual species in a class over a 1–3 min retention time window. Each species has a slightly different retention time, which enhances chances to characterize minor species. Table 1 lists only phospholipids whose structure is confirmed by at least three fragment ions, 1-MAG-like ions, and two carboxylate anions (one for ether phospholipids).

As shown in Table 1, more saturated fatty acids are always found at the sn-1 position, consistent with the typical structure of phospholipids (1). Although, the ratio of 2-MAG-like ions/1-MAG-like ions varies depending on the class of phospholipid and acyl chain composition, the difference is always significant (*P* < 0.4). The presence of structural isomers, for example, PE(16:0/20:4) and PE(20:4/16:0), also may be responsible for this variation. However, the results clearly support the use of the peak intensity of MAG-like ions to determine the position of acyl chains, as previously proposed (10, 11).

## CONCLUSIONS

Separation of classes was essential for in-depth characterization of phospholipids. Simplified MS/MS spectra by class separation allowed the detailed structural characterization of phospholipids from BHMs. We characterized various ether phospholipid species in PS, PE, and PC and phospholipid species containing odd-numbered acyl chains in CL, PG, PS, and PI. We also characterized several species of MLCLs and DLCLs by MS/MS. More importantly, we further confirmed that that MAG-like ions have the potential to be used to determine the acyl chain position. The results clearly show the power of normal-phase HPLC-MS/MS for analysis of phospholipids.

### Data availability

All data used are contained within the article.

## Supplementary Material

Supplemental Data
