# Supplementary material for: Identification of unusual phospholipids from bovine heart mitochondria by HPLC-MS/MS
Source: J Lipid Res. 2020 Sep 30;61(12):1707–19. doi: 10.1194/jlr.RA120001044 (PMC7707168; doi:10.1194/jlr.RA120001044)
Supplement: Supplemental Data [file supp_61_12_1707__index.html]

Identification of Unusual Phospholipids from Bovine Heart Mitochondria by HPLC-MS/MS — HPLC-MS/MS analysis of mitochondrial phospholipids — Identification of unusual phospholipids from bovine heart mitochondria by HPLC-MS/MS — Supplemental Data 

# Identification of unusual phospholipids from bovine heart mitochondria by HPLC-MS/MS

## Supplemental Data

- Supplementary\_figures and tables - Supplementary Material to Identification of Unusual Phospholipids from Bovine Heart Mitochondria by HPLC-MS/MS, etc..
