# Supplementary material for: Identification of unusual phospholipids from bovine heart mitochondria by HPLC-MS/MS
Source: J Lipid Res. 2020 Sep 30;61(12):1707–19. doi: 10.1194/jlr.RA120001044 (PMC7707168; doi:10.1194/jlr.RA120001044)
Supplement: Supplemental Data [file supp_RA120001044_162493_3_supp_602915_qh866z.pdf]

Supplementary Material
to

Identification of Unusual Phospholipids from Bovine Heart Mitochondria by HPLC-MS/MS

Junhwan Kim[1,2] and Charles L. Hoppel[1,2,3*]

[1]Center for Mitochondrial Diseases and Departments of [2]Pharmacology and

[3]Medicine, Case Western Reserve University, Cleveland, Ohio 44106

Running title: HPLC-MS/MS analysis of mitochondrial phospholipids

[*] Correspondence to:

Charles L. Hoppel, M.D.
Case Western Reserve University
School of Medicine
Department of Pharmacology
10900 Euclid Avenue
Cleveland, OH 44106
Ph: (216) 368-3147
fax (216) 368-5162
E-mail charles.hoppel@case.edu

Table S1. Phospholipid content in mitochondria (nmol/mg mitochondria protein)

	PE	PG	PI	CL	MLCL	PC	Total
bovine heart	248.0 (±13.9)	1.8 (±0.1)	18.2 (±0.3)	51.8 (±2.3)	0.6 (±0.0)	194.7 (±11.5)	515.0 (±17.7)
mouse heart[a]	255.4 (±26.4)	4.02 (±0.6)	16.8 (±2.0)	60.96 (±9.0)	1.0 (±0.3)	220.7 (±28.6)	558.8 (±64.7)
rat heart[a]	230.8 (±8.7)	4.4 (±0.4)	19.0 (±2.2)	48.3 (±1.7)	1.9 (±0.2)	201.7 (±17.4)	506.0 (±23.8)
rat skeletal muscle	245.4 (±11.0)	2.1 (±0.2)	19.7 (±3.5)	59.9 (±6.2)	1.1 (±0.6)	216.3 (±17.8)	544.5 (±28.3)

[a] phospholipids from subsarcolemmal mitochondria

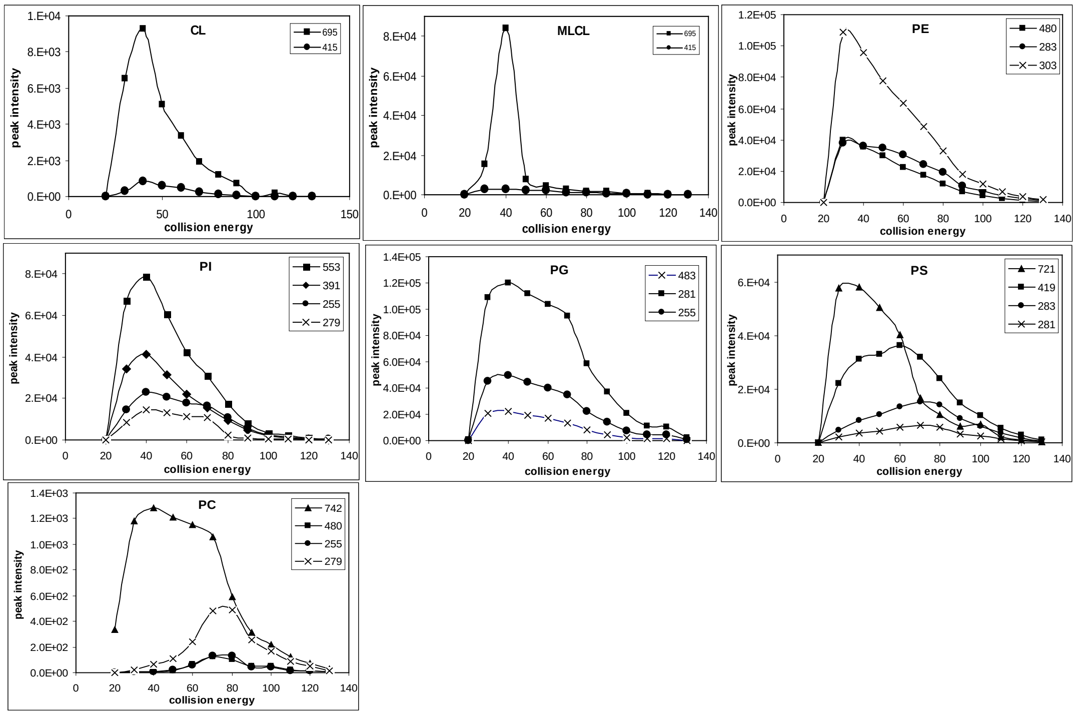

Figure S1. Intensities of fragment ions from phospholipids as a function of collision energy. Standard phospholipids were infused in eluent A and intensities of fragment ions from a specific species in each class were monitored by varying collision energy from 20 to 130 eV. (For PS and PC, the peaks at 742 and 721 were scaled 1:10 for the figure).

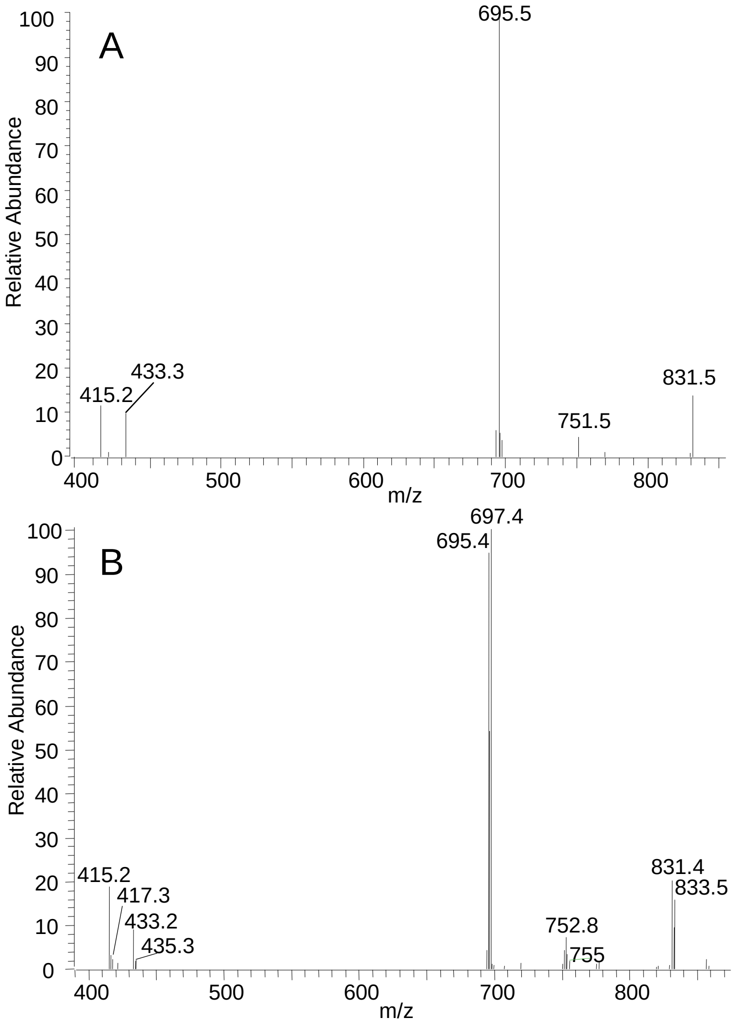

Figure S2. The MS/MS spectra of CL species resulting after collisional activation of the $[M-H]^-$ ions at m/z (A) 1448 and (B) 1450. The MS/MS peaks at 695 and 433/415 (A) and at 697, 695, 433/415, and 435/417 (B) confirm the structures as CL(18:2)$_4$ and CL(18:2)$_3$(18:1)$_1$, respectively.

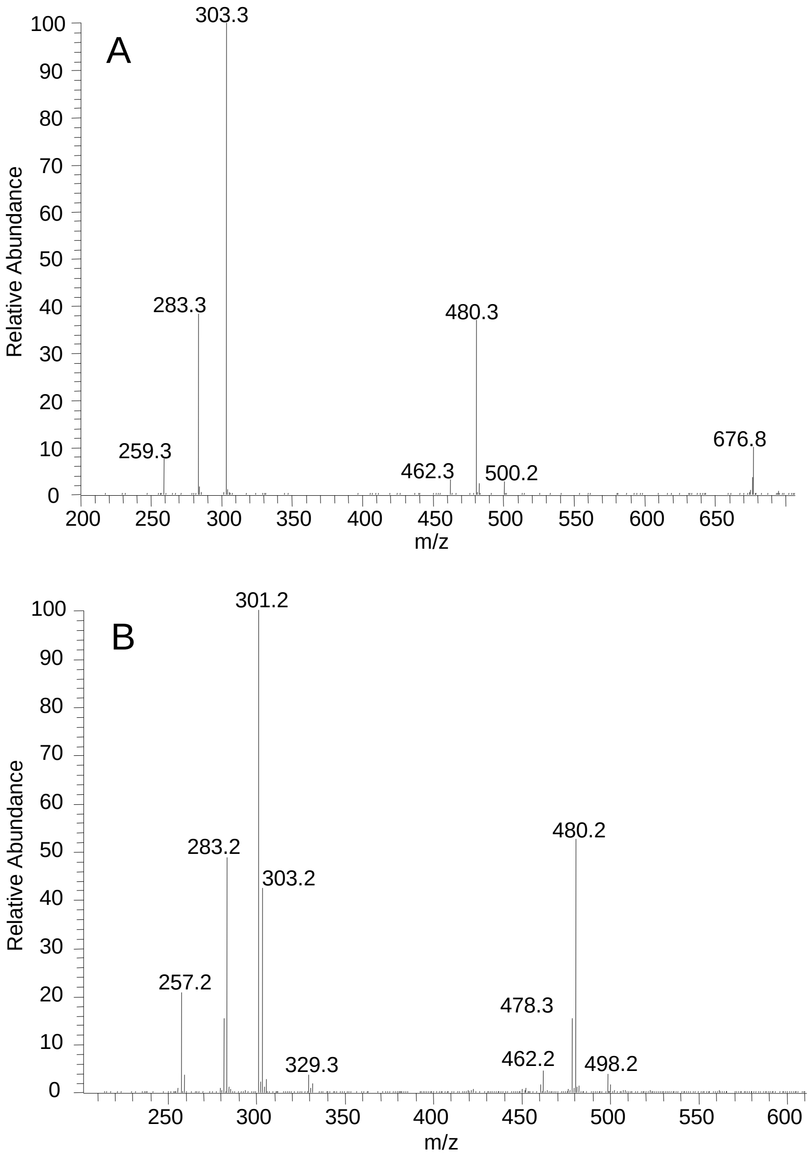

Figure S3. The MS/MS spectrum of PE molecular species resulting after collisional activation of the [M-H]$^-$ ions at m/z (A) 766.5 and (B) 764.5. The more intense peaks at 480/462 correspond to MAG1 confirm the structure as PE(18:0)(20:4) (A) and the peaks at 480/462 and 478 corresponding to MAG1 confirm the structures as PE(18:0)(20:5) and PE(18:1)(20:4) (B).

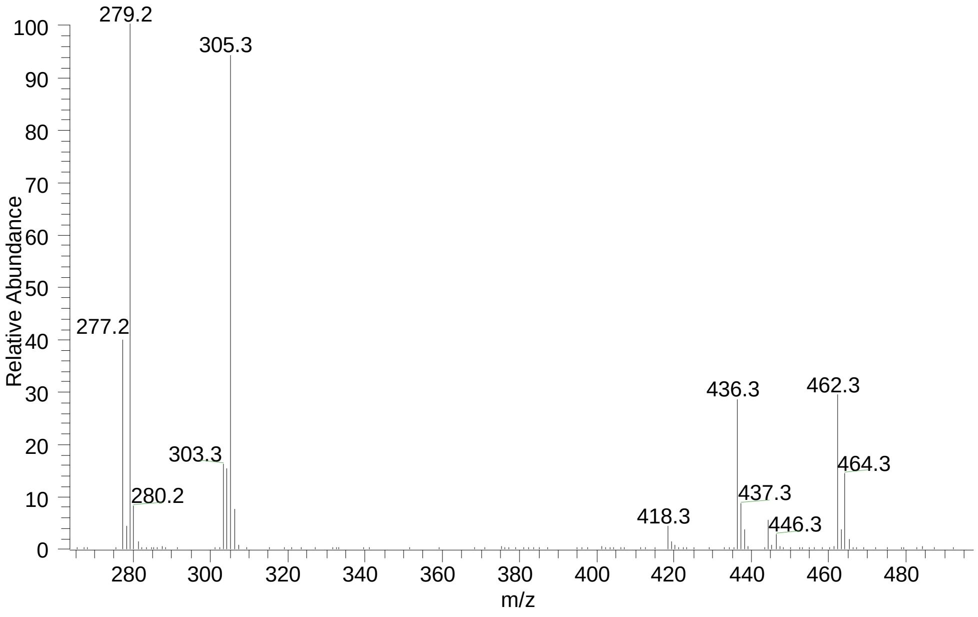

Figure S4. The MS/MS spectrum of a PEp molecular species resulting after collisional activation of the $[M-H]^-$ ion at m/z 724.5. The peaks at 436/418, 462/444, and 464 and matching acyl chains at the sn-2 positions, as shown at 305, 279, and 277, confirm the structures of three major species as PEp(16:1)(20:4), PEp(18:2)(18:2), and PEp(18:1)(18:3).

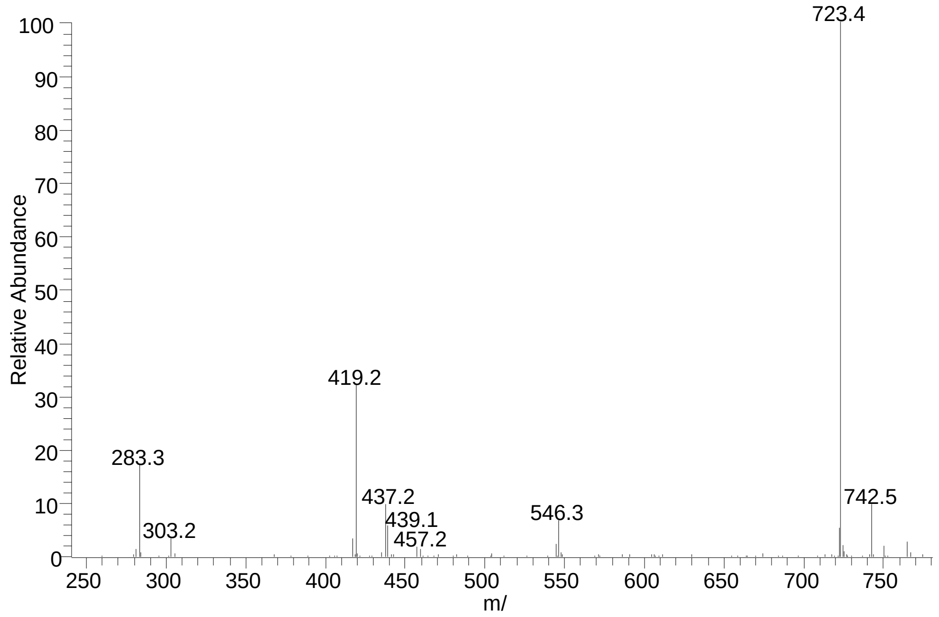

Figure S5. The MS/MS spectrum of a PS molecular species resulting after collisional activation of the $[M-H]^-$ ion at m/z 810.5. The more intense peaks at 437/419 corresponding to MAG1 than the peaks at 457/439 corresponding to MAG2 confirm the structure as PS(18:0)(20:4).
